# Small Volume Dissolution Testing as a Powerful Method during Pharmaceutical Development

**DOI:** 10.3390/pharmaceutics2040351

**Published:** 2010-11-01

**Authors:** Scheubel Emmanuel, Lindenberg Marc, Beyssac Eric, Cardot Jean-Michel

**Affiliations:** 1 Biopharmaceutical department, Faculty of Pharmacy, University of Auvergne 28 Place H. Dunant, BP 38, 63001 Clermont-Ferrand, France; E-Mails: eric.beyssac@u-clermont1.fr (B.E.) ; j-michel.cardot@u-clermont1.fr (C.J.-M.); 2 Galenical & Analytical Development, Pharmaceutical Division, F. Hoffmann-La Roche Ltd., CH-4070, Basel, Switzerland; E-Mail: marc.lindenberg@roche.com (L.M.)

**Keywords:** Dissolution, Small volume, Discrimination, Screening, Quality By Design

## Abstract

Standard compendia dissolution apparatus are the first choice for development of new dissolution methods. Nevertheless, limitations coming from the amount of material available, analytical sensitivity, lack of discrimination or biorelevance may warrant the use of non compendial methods. In this regard, the use of small volume dissolution methods offers strong advantages. The present study aims primarily to evaluate the dissolution performance of various drug products having different release mechanisms, using commercially available small volume USP2 dissolution equipment.

The present series of tests indicate that the small volume dissolution is a useful tool for the characterization of immediate release drug product. Depending on the release mechanism, different speed factors are proposed to mimic common one liter vessel performance. In addition, by increasing the discriminating power of the dissolution method, it potentially improves know how about formulations and on typical events which are evaluated during pharmaceutical development such as ageing or scale–up. In this regard, small volume dissolution is a method of choice in case of screening for critical quality attributes of rapidly dissolving tablets, where it is often difficult to detect differences using standard working conditions.

## 1. Introduction

Dissolution testing is a core performance test in pharmaceutical development and quality control. Dissolution testing has more and more evolved to establish relationships with *in vivo* performance or with manufacturing Critical Quality Attributes (CQA) in the scope of Quality by Design (QbD)[1].. The overall goal is to better control product performance within the life cycle of a product. For this purpose, the use of the classical USP dissolution working conditions using a one liter vessel with basket (respectively USP1) and paddle (respectively USP2) are well established [2,3] and are used as the first choice for development of a new dissolution method.

Nevertheless, limitations coming from the amount of material available, analytical sensitivity, lack of discrimination or biorelevance may warrant the use of non compendial methods. In particular, in early phase development, during screening of drug candidates, formulation is often developed for studies in animals and dissolution should be ideally conducted using media simulating the gastrointestinal environment as well as in volumes in line with the animal physiology [4]. Another case in which a classical method is not well suited is for low dose drugs or if the analytical method is not sensitive enough to detect the amount of dissolved drug precisely due to low concentration of the drug in the formulation [5]. To overcome those problems the concept of small-volume dissolution arose recently due to the possibility of using smaller sample sizes and smaller volumes of media, offering various advantages in view of substance and material consumption [6] and can serve as a valuable tool for dosage form screening [7] or formulation selection in animals.

The present study aims primarily to evaluate the potential of commercially available small volume USP2 dissolution equipment for the dissolution of solid drug product. This miniaturized vessel/paddle equipment can be easily fitted, without hardware change or adaptation, on a classical USP2 system. For this purpose, different kinds of dissolution release mechanisms for solid drug products; immediate release (IR), extended release (ER) as well as low dose tablets, were screened using both standard (one liter) and small volume dissolution setup. Working conditions to achieve the same dissolution performance for both tests were sought using the small volume equipment. Attempts to generalize these dissolution working conditions for new products are discussed. The discriminating power of the method is stressed through one example of IR tablets by comparing the contribution of the small vessel dissolution on typical events faced during development such as aging and scale–up *versus* compendial apparatus.

## 2. Experimental Section

### 2.1. Materials

Phosphate buffer, sodium chloride, 37% hydrochloric acid (fuming), 85% ortho-phosphoric acid, ethanol (99.9%) as well as HPLC grade methanol were purchased from Merck (Darmstadt, Germany). Water was obtained from a Milli-Q (Millipore, Milford, MA, USA) water purification system. For all tests, GR grade material was used.

### 2.2. Methods

**Figure 1:**
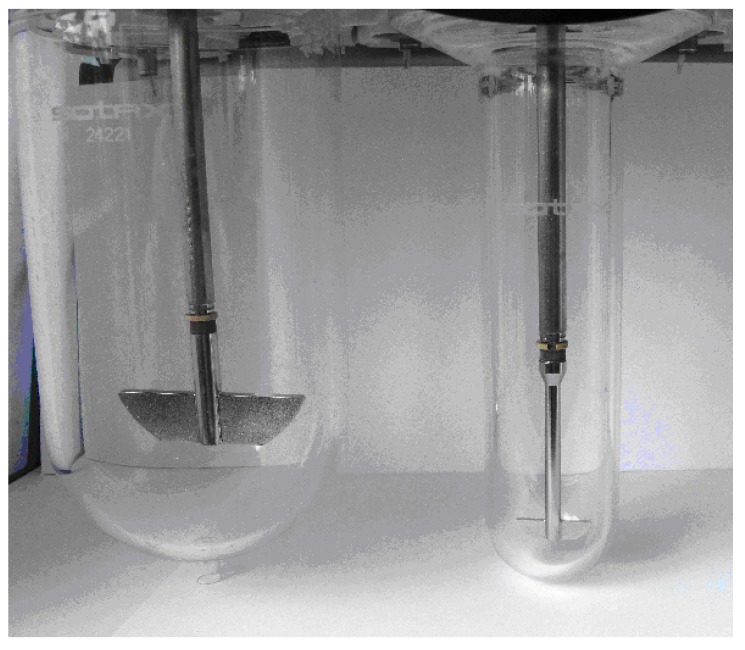
A small volume vessel equipped with small paddle (right side) and the compendial one liter vessel with paddle (left).

Dissolution experiments were performed using a Sotax AT7 smart apparatus (Sotax, Allschwill, CH). The small volume vessel is based on the USP one liter vessel setup, the size was reduced to be used with 50 mL to 200 mL of dissolution medium with an internal diameter of 40 mm. The Sotax small volume vessel is a single device and offers the advantage to be installed directly on existing equipment. A small paddle blade of 29 mm length fitted at 10 mm from bottom of the vessel is used. An overview of the small volume set up is presented Figure 1 and the different sizes of the small volume equipments are listed in Table 1. The investigations were conducted in 150 mL, working conditions that allow providing sink condition for all tested products. 

The aim of the series of tests was to establish a relationship between the reference one liter vessel method (using 900 mL or 500 mL of media) and the small vessel accessories (composed of small vessel and small paddle). For this purpose, the rotation speed of the small vessel system was varied from 50 rpm up to 150 rpm to evaluate the speed factor (sf) between both methods. All the tests were performed in triplicate for screening purposes and with 6 units during the evaluation of scale–up and ageing with one example in order to confirm the early findings and assess the potential of the method during development. An overview of the dissolution working conditions for the classical one liter dissolution method is presented Table 2. The samples were collected semi automatically, filtrated and measured according to USP or by validated UV or HPLC methods. For all tests the same dissolution system was used.

**Table 1:** Dissolution – Difference in Dimension (mm) of the small and USP Vessels and Paddle.

	USP one liter vessel	Small volume Apparatus
**Vessel**		
Height	168 ± 8	185
Internal diameter	102 ± 4	40
**Paddle **		
Blade Upper chord	74.0 ± 0.5	29
Blade Lower chord	42.0 ± 1.0	18
Height	19.0 ± 1.0	7.5
Distance from the bottom	25 ± 2	10

### 2.3. Model compounds

Five different products exhibiting different type of release rates were chosen. Both Performance Verification Test tablets (prednisone [8] and salicylic acid [9], disintegrating and non disintegrating tablets respectively) were bought at USP, Rockville USA. Experimental IR formulations and ER tablet formulations were supplied by Roche Pharmaceutical Research department, Basel, CH. The ER tablets formulations were produced by wet granulation using different amounts of HPMC to achieve four hour (ER4H) and eight hour (ER8H) release profiles. The IR formulations are either immediate release, low dose tablet (IR(1)) or a very rapidly dissolving tablet IR(2), both exhibiting 85% dissolved within 15 minutes in classical conditions. 

The Active Pharmaceutical Ingredient (API)’s of these five drug products exhibit high or low solubility according to the biopharmaceutical classification system (BCS) [10]. However, the medium chosen during these investigations were set up in order to reach sink conditions in 150 ml. For each product, the same medium was used for the one liter and for the small vessel. An overview of the tablet types and properties is listed in Table 2.

**Table 2:** Overview of the tablets and release mechanisms tested using both dissolution methods.

Product	Strength (mg)	BCSclass	Dissolution method with one liter vessel		Releasemechanism	Tablets types
Prednisone Batch :POE203	10 mg	1	500 mL	Paddle 50 rpm	IR	Disintegrating
Salicylic acid Batch :Q0D200	300 mg	3	900 mL	Paddle 100 rpm	ER	Non-disintegrating
ER4H / ER8H	1 mg	2*	500 mL	Paddle 50 rpm	ER	Erosion-Diffusion
IR(1)	0.075 mg	1	500 mL	Paddle 50 rpm	IR	Disintegrating
IR(2)	50 mg	2*	900 mL	Paddle 50 rpm	IR	Disintegrating

ER = Extended Release; IR = Immediate Release; *Active principle having pH dependent solubility. Medium was chosen to provide sink condition in 150 mL.

For IR(2), comparison after storage for three months at 25 °C/60% relative humidity (r.h.) and 40 °C/75% r.h. according to ICH conditions and after scale–up (8 kg to 15 kg) were performed using both methods. 

### 2.4. In vitro dissolution test comparison

For the screening purpose of the study, in addition to a visual comparison of the dissolution profiles, where the shape and the plateau of the curves were estimated, the closeness of the profiles was assessed by calculating the ratio of percent dissolved at each time point according to equation 1 and the mean ratio for all sampling points was assessed using equation 2. 


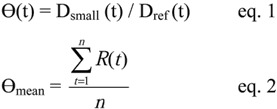


ϴ(t) represents the ratio at time t, D_small_ the percent dissolved for the small volume method and D_ref_ the percent dissolved for the reference method (so called one liter). ϴ_mean_ represents mean of the ϴ(t).

A ϴ_mean_ close to one is sought with a ratio stable all along the profile. ϴ_mean_ above one would mean that the profiles have the tendency to be faster than the reference. ϴ_mean_ below one would mean that the profiles have the tendency to be slower than the reference. Applying such a ratio assumes that the dissolution curves exhibit similar profiles with only a difference in the rate of dissolution. The f2 factors [11] were calculated on the mean dissolution values as an additional factor to the ϴ_mean_. 

## 3. Results and Discussion

**Figure 2:**
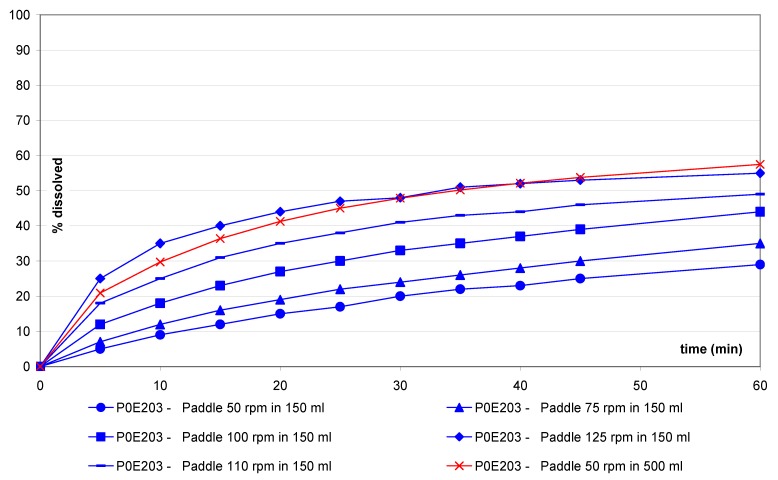
Dissolution profiles for prednisone tablets with small vessel accessories *versus* USP method with one liter vessel.

**Figure 3:**
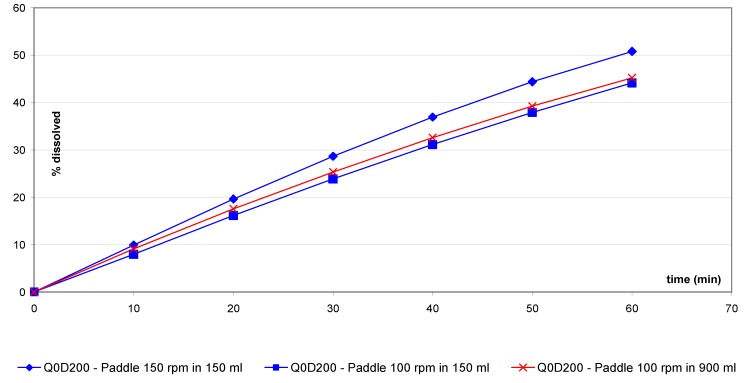
Dissolution profiles for salicylic acid tablets with small vessel accessories *versus* USP method with one liter vessel.

**Figure 4:**
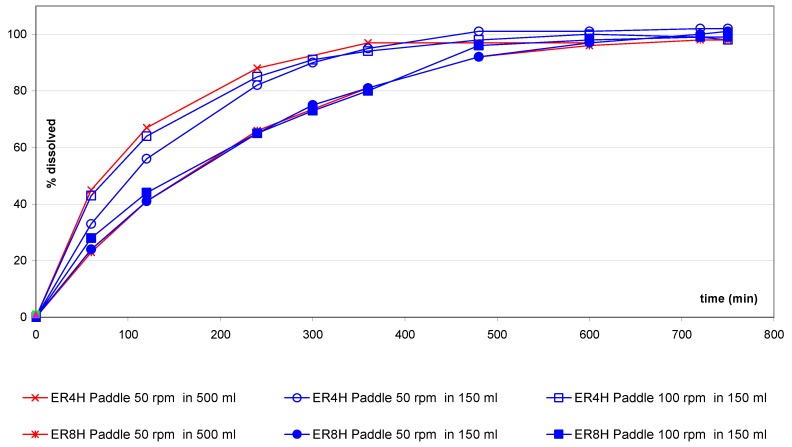
ER4H and ER8H tablets: comparison of small vessel accessories *versus* one liter vessel.

**Figure 5:**
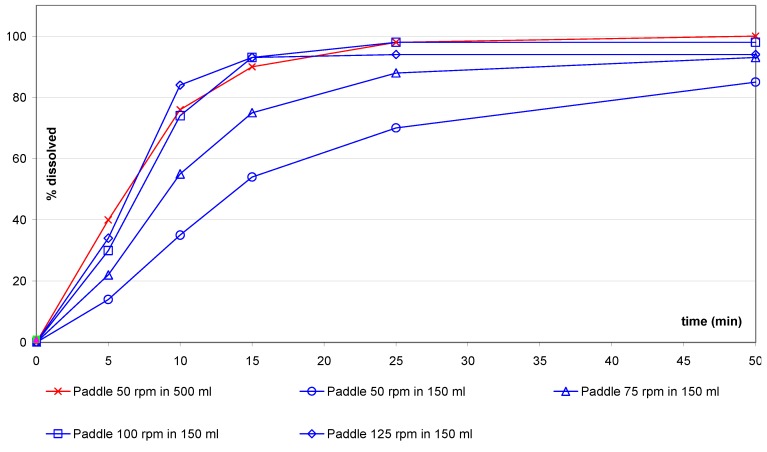
IR(1) tablets: comparison of small vessel accessories *versus* one liter vessel.

**Figure 6:**
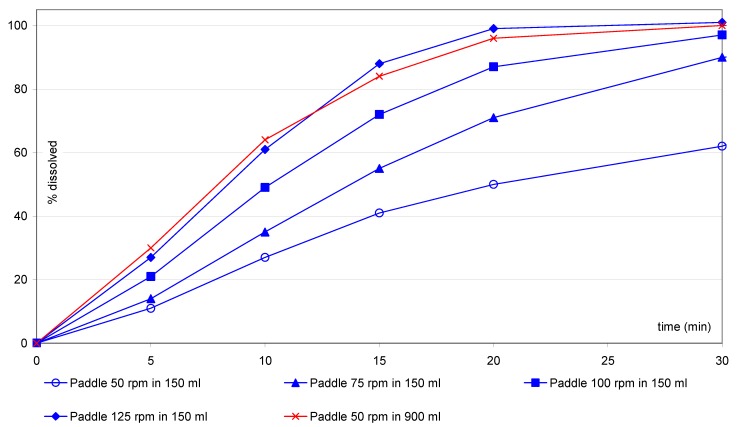
IR(2) tablets: comparison of small vessel accessories *versus* one liter vessel.

**Figure 7:**
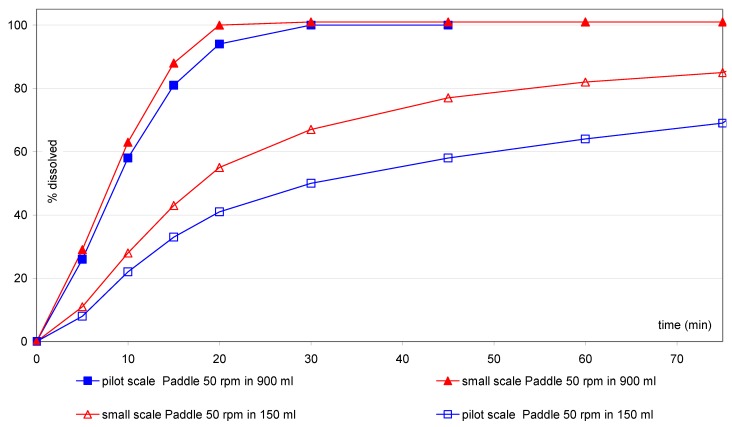
IR(2) tablets: comparison after scale–up using small vessel accessories.

**Figure 8:**
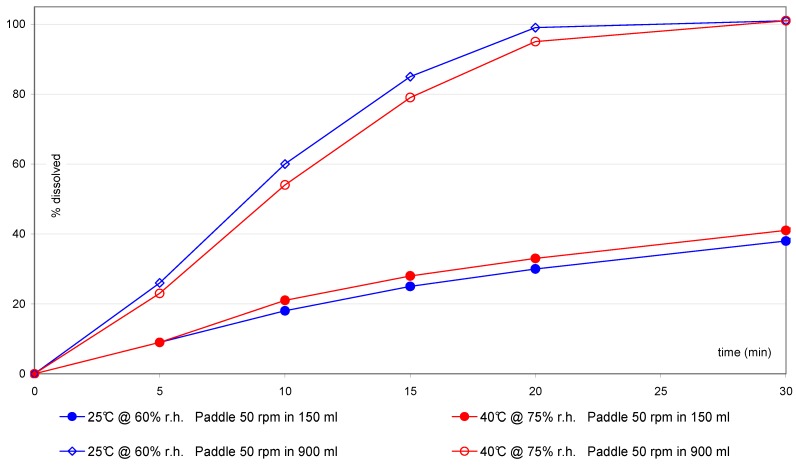
IR(2) tablets: comparison after storage using small vessel accessories.

Figure 2, Figure 3, Figure 4, Figure 5, Figure 6, Figure 7 and Figure 8 and show the mean dissolution profiles of all tested variants and Table 3 shows the mean of the ratios. Similar findings were found for the ratios and the f2 factors. No coning or mounting was observed using the small volume vessel except for the prednisone disintegrating tablets, which was also seen for the one liter vessel. Similar curve shapes were observed for prednisone, salicylic acid as well as for ER tablets. Slightly different curves shape and time to reach the plateau were observed for the IR(1) and IR(2) tablets. For all dissolution experiments, the observed standard deviations (SD) are low (maximum of 6% at first sampling point and below 5% for the next sampling points). The SD are similar for both small volume and one liter methods through the entire profiles.

**Table 3:** Mean of ratio (ϴ_mean_) percent dissolved between small and one liter dissolution at different rotation speeds. Best values are in bold.

Product	Reference Method	Small vessel rotation speed					
		50 rpm	75 rpm	100 rpm	110 rpm	125 pm	150 rpm
Prednisone	Paddle 50 rpm	0.39	0.48	0.67	0.85*	1.05*	-
Salicylic acid	Paddle 100 rpm	-	-	0.76*	-	-	0.96*
ER4H	Paddle 50 rpm	0.93*	-	0.98*	-	-	-
ER8H	Paddle 50 rpm	1.01*	-	1.05*	-	-	-
IR(1)	Paddle 50 rpm	0.59	0.79	0.95*	-	0.98*	-
IR(2)	Paddle 50 rpm	0.57	0.71	0.86	-	0.99*	-

* indicates the f2 factors between small and one liter vessel with a value above 50.

When using an identical rotation speed, the small volume vessels showed a lower percent of drug dissolved than the one liter vessel for most of the methods except for the slowest ER8H using the paddle at 50 rpm. 

For prednisone (Figure 2), a small vessel/paddle at 125 rpm resulted in a similar profile compared to the USP paddle 50 rpm method. This corresponds to a speed factor (sf) of 2.5 (sf = 2.5). 

For salicylic acid non-disintegrating tablets (Figure 3), a small vessel/paddle at 150 rpm results in a similar profile to the USP paddle 100 rpm method (sf = 1.5). 

For the extended release tablets ER4H and ER8H (Figure 4), the impact of the small vessel/paddle setup is less pronounced. By varying the rotation speed from 50 to 100 rpm, similar profiles can be observed and the ratios remain very close.

For the IR(1) tablets (Figure 5), both motion speeds of 100 rpm and 125 rpm when using the small vessel/paddle resulted in a similar profile to the one liter method with paddle at 50 rpm (sf = 2.5). 

For the IR(2) tablets (Figure 6), use of the small vessel/paddle at 125 rpm resulted in a similar profile to the one liter method at paddle 50 rpm method (sf = 2.5). 

The comparison of samples after storage (Figure 7) does not show a difference, whereas after scale–up (Figure 8) a new trend is visible only using the small vessel at 50 rpm.

All those results are summarized in Table 4.

**Table 4:** Found rotation speed factors using small vessel *versus* one liter vessel to reach the same performance.

Tablet type	Product	Dissolution method	Rotation speed using one liter vessel	Rotation speed using small vessel	*Rotation speed Factor (sf)*
disintegrating	Prednisone	Paddle	50	125	2.5
disintegrating	IR(1)	Paddle	50	125	2.5
disintegrating	IR(2)	Paddle	50	125	2.5
Non-disintegrating	Salicylic acid	Paddle	100	150	1.5
Non disintegrating	ER4H	Paddle	50	50-100	1-2
Non disintegrating	ER8H	Paddle	50	50-100	1-2

These investigations clearly showed that using the small vessel set up, equivalent or higher rotational speeds are necessary to obtain similar dissolution rates when compared to the one liter vessel. Speed factors from 1 to 2.5 have been observed (see Table 4). 

A theoretical calculation of the rotation speed needed for the small paddle to reach the velocity of the large paddle at 50 rpm was performed based on the differences of the paddle sizes (Table 5) [12]. A corresponding rotation speed of 121 to 129 rpm was found. This difference corresponds to a speed factor of 2.5.

**Table 5:** Theoretical calculation of hydrodynamics difference between small paddle and large paddle.

		Equation	Length on top of the paddle		Length on bottom of the paddle	
			small	large	small	large
Rotation/rpm	R		100.00	50.00	100.00	50.00
Frequency/Hz	F	R/60	1.67	0.83	1.67	0.83
Periodicity/s	T	1/F	0.60	1.20	0.60	1.20
Angular velocity/rad·s^-1^	W	2pi/T	10.51	5.25	10.51	5.25
1/2 lenght/mm	R		14.50	37.25	8.70	21.00
Linear speed on top of the paddle/cm·s^-1^	V	R*W	152.33	195.66	91.40	110.31
Calculation of the angular velocity for the small paddle/rad·s^-1^	W		13.49		12.68	
Periodicity/s	T		0.47		0.50	
Frequency/Hz	F		2.15		2.02	
			128.86	≥ 129	121.07	≥ 121

A speed factor of 1.5 was observed for salicylic acid tablets and 1 to 2 for the ER formulations. A speed factor of 2.5 was observed for the IR formulations (prednisone , IR(1) and IR(2)) indicating that the working conditions to obtain the performance of one liter vessels in small vessels clearly depend on the type of release mechanism. 

In the case of the fast dissolving IR formulation, as presented in this paper, one of the main factors to take into account beside the intrinsic properties of the API (e.g., solubility) is the rate of renewal of the dissolution media in contact with the API. Based on Noyes Whitney equation [13] and diffusion layer term [14,15], it is directly in relation to the rotation speed of the dissolution method.

In case of the salicylic acid tablets or the ER formulations, the limiting factor is not driven only by dissolution properties of the API but rather by the design of the formulation (e.g., erosion/diffusion [16]) and, therefore the characteristics of the formulation are less dependent on the renewal of the media as soon as this renewal is faster than the release rate [17,18,19] This phenomenon is emphasized *in vitro* for the longer releasing tablets. In our example for the ER8H, no difference could be observed between both methods and that independently of the rotation speed in small vessels. Diffusion controlled tablets would then not be impacted by the hydrodynamics [20] and the speed factor may come close to 1.

For tablets impacted by small volumes, a higher discriminating power may be expected by measuring of rapidly dissolving tablets using a small vessel at 50 rpm or less. In this case, 50 rpm in a small vessel would correspond approximately to 20 rpm (50 rpm divided by sf 2.5) in a one liter vessel, which would be out of the range of standard performance verification test of the apparatus. 

Based on this observation, further investigations were tried with the IR(2) tablets. At 50 rpm with the small vessel/paddle, the differences after manufacturing scale–up are more pronounced than with the one liter vessel (Figure 7), whereas no significant change can be observed after storage under different temperatures (Figure 8). These differences highlight a possible change of the intrinsic quality of the tablets after manufacturing scale–up, whereas the product seems to be very stable after three months storage even under stress storage conditions and using the most discriminating dissolution method.

The significance of the observed difference does not mean that a change in *in vivo* performance should be expected, the profiles remain very rapidly dissolving and both tablets should be completely dissolved before gastric emptying [21]. However, this difference points out a change in the tablets’ properties after scale–up and further investigations into manufacturing parameters and resulting solid state properties may be initiated. In this regard, the small vessel dissolution method supports a better process understanding and is in line with a QbD approach. 

Results from the present series of tests indicated that the small paddle apparatus might be a useful tool in characterizing drug release profiles under standard test conditions, mainly to IR and disintegrating tablets as it was shown to be more discriminant. 

Takano et al [22] showed that small volumes can also be applied for low soluble molecules even under non sink conditions

During development of the small volume method, it is important to take into account that the current small or low volume vessels are non compendial. The commercially available vessels are well defined [23] but there are still differences from supplier to supplier. It was demonstrated that differences in the actual compendia apparatuses existed between suppliers even if within the standardized dimensions and that those differences marginally affected the results [24]. In case of small volume vessels there is no currently fixed dimension between suppliers. This means that each investigation should be carry out specifically and that transfer is more complicated than using the classical pharmacopeia one liter vessel. 

The discriminating power of the small volume method seems more pronounced for IR compared to ER formulations. It is therefore recommended to systematically integrate small volume methods in the screening of new methods for IR formulation. 

## 4. Conclusion

This limited set of data clearly showed that the small volume apparatus is a useful tool in the characterization of solid drug product dissolution profiles. It can be easily installed in a standard laboratory, it uses standardized working conditions and can be set up to fit to the common one liter vessel performance when the dissolution method is not rugged enough for instance with an analytical method having an improper sensitivity. In addition beside the advantage of using smaller volumes of media, it potentially allows to expand the discriminating power of a method by applying gentle agitation which is particularly important for IR and disintegration tablets. Only two IR tablets within sink conditions were exemplified and further tests should be initiated to consolidate these first outcomes. Nevertheless these data taken as a starting point showed that this approach improves know how about formulations, the process and is a method of choice instances of screening for CQA of rapidly dissolving tablets where it is often difficult to detect difference using standard working conditions.
